# Preparation and characterization of methacrylated gelatin/bacterial cellulose composite hydrogels for cartilage tissue engineering

**DOI:** 10.1093/rb/rbz050

**Published:** 2019-12-19

**Authors:** Liling Gu, Tao Li, Xiongbo Song, Xianteng Yang, Senlei Li, Long Chen, Pingju Liu, Xiaoyuan Gong, Cheng Chen, Li Sun

**Affiliations:** 1 Medical College, Guizhou University, Guiyang 550025, China; 2 Department of Rehabilitation, Guizhou Provincial People’s Hospital, Guiyang 550002, China; 3 Center for Joint Surgery, Southwest Hospital, Third Military Medical University (Army Medical University), Chongqing 400038, China; 4 Department of Orthopedics, Guizhou Provincial People’s Hospital, Guiyang 550002, China; 5 Zunyi Traditional Chinese Medicine Hospital, Zunyi 563099, China

**Keywords:** methacrylated gelatin, bacterial cellulose, hydrogel, cartilage tissue engineering

## Abstract

Methacrylated gelatin (GelMA)/bacterial cellulose (BC) composite hydrogels have been successfully prepared by immersing BC particles in GelMA solution followed by photo-crosslinking. The morphology of GelMA/BC hydrogel was examined by scanning electron microscopy and compared with pure GelMA. The hydrogels had very well interconnected porous network structure, and the pore size decreased from 200 to 10 µm with the increase of BC content. The composite hydrogels were also characterized by swelling experiment, X-ray diffraction, thermogravimetric analysis, rheology experiment and compressive test. The composite hydrogels showed significantly improved mechanical properties compared with pure GelMA. In addition, the biocompatility of composite hydrogels were preliminarily evaluated using human articular chondrocytes. The cells encapsulated within the composite hydrogels for 7 days proliferated and maintained the chondrocytic phenotype. Thus, the GelMA/BC composite hydrogels might be useful for cartilage tissue engineering.

## Introduction

Articular cartilage is a highly specialized connective tissue that diffuses the load and lowers the friction within synovial joints [1]. It has no blood supply and has a very low cellularity [2]. Such unique nature of cartilage makes cartilage repair a daunting task in the field of clinical orthopedics and sports medicine. To improve the outcome of cartilage repair and regeneration, tissue engineering strategy has been studied extensively both *in vitro* and *in vivo* [3, 4].

Bacterial cellulose (BC), a natural polysaccharide, has shown promise as a scaffold for cartilage tissue engineering [5–7]. This nanostructured material, produced by *Acetobacter xylimum*, shows high crystallinity, high water absorption capacity and good mechanical properties such as toughness, resilience and flexibility [8, 9]. In 2005, Svensson *et al*. [10] first proposed the use of BC scaffold for cartilage tissue engineering, as BC scaffold well-supported the proliferation of chondrocytes. However, the relatively dense nanostructure of BC limits cell ingrowth and migration [11–13]. To overcome this problem, several strategies have been applied to improve cell ingrowth in BC scaffolds by adjusting pore size and pore interconnectivity during synthesis of BC [14, 15], through laser ablation [16, 17] or freeze-dry processing [18]. In addition, the BC based composites have been investigated for better cell ingrowth and tissue regeneration. For example, BC network changes in the presence of type-I collagen [19], and this BC/collagen composite shows potential for bone tissue engineering [20]. Other BC composites, such as BC/chitosan [21] and BC/silk fibroin [22], also show a better support for fibroblast adhesion than pure BC scaffold. However, BC composites for cartilage tissue engineering have been less studied.

Gelatin is derived from collagen hydrolysis and is widely used in cartilage tissue engineering [23–25]. Recently, methacrylated gelatin (GelMA) has gained increased attention, as it combines the bioactivity of gelatin and the physicochemical tailorability of photo-crosslinking [26–28]. In this study, our aim was to develop a composite based on BC and GelMA for cartilage tissue engineering application. The GelMA/BC hydrogel could be prepared under UV irradiation, without the use of cytotoxic chemical cross-linking agent. Moreover, this hydrogel is injectable to allow the repair of articular cartilage defects of different shapes. To this end, GelMA/BC hydrogels with different BC concentrations were fabricated and characterized; the cytocompatibility of the composite hydrogel was also assessed by encapsulation of human articular chondrocytes.

## Materials and methods

### Preparation of GelMA

GelMA was obtained according to a previous protocol with minor modifications [29]. In brief, Type A porcine skin gelatin (Sigma, USA) solution with a concentration of 10% was prepared using PBS at 50°C. Methacrylic anhydride (MA; J&K Chemical, China) was added dropwise to this gelatin solution to reach a final MA concentration of 20%. The mixture was stirred at 50°C for 3 h, then the GelMA solution was dialyzed against Milli-Q water for 1 week in dialysis bag (3500 kDa) at 40°C. Then, the freeze-dried product was stored at 4°C until further use. The structure of GelMA was characterized by proton nuclear magnetic resonance (^1^H NMR) spectroscopy and the degree of substitution (DS) of MA on gelatin was calculated.

### Preparation of BC particles

The BC membranes (Hewu Biological Technology, China) were boiled in NaOH solution (0.1 M) for 1 h, and then rinsed repeatedly with Milli-Q water to neutral PH. The purified BC membranes were cut into small pieces and then broken into particles by a homogenizer. The solid content of BC particles in suspension was determined by dry weighing method. To measure the mean hydrodynamic diameter and size distribution of the BC particles, dynamic light scattering (DLS) of particles in water was performed with Malvern Zetasizer Nano ZS instrument (Malvern, UK) at room temperature (RT). The samples were dispersed on glass slide and dried at 25°C, scanning electron microscopy (SEM) was then conducted using S-3400NIISEM (Hitachi, Japan) operated at 2 kV accelerating voltage to observe the particle morphology.

### Fabrication of GelMA/BC composite hydrogels

The lithium phenyl(2,4,6-trimethylbenzoyl)phosphinate (LAP; Sigma, USA) was used as a water-soluble UV photoinitiator to form the GelMA/BC composite hydrogels by photopolymerization. In brief, GelMA was dissolved in Milli-Q water with final concentration at 16 mg/ml and mixed with the different concentration of BC suspensions (2, 4, 8 and 16 mg/ml) with the ratio of 1:1 (v/v). LAP solution with a concentration of 1 mg/ml was added to the mixed solution of GelMA and BC particles in a volume ratio of 1:100. Then, the GelMA and BC particles mixed solution was exposed to UV light (365 nm, 5 W) for 2 min after it was added into a cylinder mold. Different groups of GelMA/BC composite hydrogels were named 0BC (control), 1BC (1 mg/ml), 2BC (2 mg/ml), 4BC (4 mg/ml) and 8BC (8 mg/ml) according to the concentration of BC particles, respectively.

### Characterization of the GelMA/BC composite hydrogels

#### Equilibrium water uptake

The water uptake ability of the GelMA/BC composite hydrogels was detected in Milli-Q water by dry weighing method. The lyophilized hydrogels were weighed and recorded as W_0_, and then immersed in Milli-Q water to reach a swelling equilibrium at 37°C. The hydrogels which had been swollen and balanced were taken out from the Milli-Q water, and the moisture on the surface of the gel was quickly absorbed by the filter paper, and then weighed and recorded as W_*t*_. The water uptake ability of the GelMA/BC composite hydrogels was calculated by the following formula (W_*t*_ − W_0_)/W_*t*_ × 100%.

#### X-ray diffraction

The crystal structures of GelMA/BC composite hydrogel were characterized by X-ray diffraction (XRD). The prepared hydrogels were rapidly frozen with liquid nitrogen, then freeze-dried, and the obtained lyophilized hydrogels were crushed into powder. The XRD results were carried out using an X'Pert Rro MPD powder multi-function X-ray diffractometer (PANalytical, Netherlands) under the conditions of Cu Kα radiation, with a radiation intensity of 40 mA, 40 kV and a scanning speed of 6°/min. The diffraction angle (2*θ*) data were obtained from scan results between 5° and 60°, and the data were analyzed using JADE6 software (MDI, USA).

#### Differential scanning calorimetry

The melting point of GelMA/BC composite hydrogel was evaluated by differential scanning calorimetry (DSC). The sample preparation procedure was the same as for XRD. DSC results were obtained on a 1600LF thermal gravimetric/differential scanning calorimetry (TGA/DSC1) instrument (Mettler Toledo, Switzerland), with a heat rate of 2°C/min under a nitrogen gas flow of 50 ml/min.

#### Morphology characterization

The morphology of GelMA/BC composite hydrogel was characterized using SEM, operating at 2 kV accelerating voltage. Samples were prepared by cutting the lyophilized hydrogel and spraying with gold. The morphology of the sections was viewed using Hitachi S-3400NIISEM.

#### Rheological measurements

The rheological property of GelMA/BC composite hydrogel was characterized by DHR-1 rheometer (TA Instruments, USA). Parallel plate was used with a plate diameter of 20 mm and a plate gap of 500 nm. Under the oscillating mode, the strain was set as 1% and the scanning frequency was between 0.1 and 10 Hz.

#### Compression test

The mechanical property of GelMA/BC composite hydrogel was tested by Instron 5969 (Instron Instruments, USA). The height and diameter of the hydrogel were measured and the sample was first treated with a preload force of 0.06 N load cell, and then under constant force of 2 mm/min until the gel ruptured. The compressive moduli were calculated from the data of the obtained stress-strain curve between 10% and 20% strain.

### Cell culture in hydrogels

#### Isolation of human osteoarthritic chondrocytes

Cartilage samples were obtained intraoperatively from patients undergoing total knee arthroplasties with approval from Ethics Committee of Southwest Hospital (Chongqing, China). Chondrocytes were isolated according to our previous protocol [30]. In brief, cartilage pieces were digested with 0.2% type II collagenase (C6885, Sigma, USA) overnight at 37°C. Then, the cell suspension was filtered using a 40-μm cell strainer, and the cells were centrifuged (400 g for 5 min), and resuspended in high glucose DMEM (Gibco, USA) supplemented with 10% fetal bovine serum (FBS; Hyclone, USA) and 1% penicillin/streptomycin. Finally, cells were plated in T-75 flasks and incubated in a humidified atmosphere of 5% CO_2_ at 37°C. The medium was changed every 2–3 days. Only cells at passage 1 were used in our study to avoid phenotype loss.

#### Cell encapsulation and 3D culture

Chondrocytes were suspended in GelMA solution at a density of 1 × 10^6^ cells/ml. The GelMA/cell suspension was mixed with BC suspensions, and LAP (1 mg/ml) was added to the mixed solution. The mixed solution was then added into a 24-well plate, followed by UV light exposure (365 nm, 5 W) for 2 min to form the hydrogel. Then, high glucose DMEM supplemented with 10% FBS and 1% penicillin/streptomycin was added into each well. The medium was changed every 2 days.

#### Cell viability

To evaluate the viability of chondrocytes in GelMA/BC composite hydrogels, a Live/Dead staining kit (Yeasen) was used. In brief, after 1, 7 and 14 days of culture, chondrocyte-encapsulated hydrogels were incubated with 2 µM Calcein-AM and 4.5 µM propidium iodine (PI) for 15 min at 37°C. Labeled cells were visualized using a confocal microscope (IX71, Olympus, Japan).

#### Immunofluorescence staining

Samples were washed with PBS and fixed with 4% formaldehyde for 15 min at RT. Then, cells were treated with Triton X-100 (Beyotime) for 10 min at RT, and blocked 2 h with Blocking Buffer (Beyotime) at RT followed by incubation with primary antibodies: Mouse Anti-Collagen II (ab185430, 1:200; Abcam), Mouse Anti-Aggrecan (ab3778, 1:200; Abcam), Rabbit Anti-SOX9 (ab185230, 1:200; Abcam) for overnight at 4°C. Next, the cells were washed three times with PBST, incubated with secondary antibody (Goat anti-Rabbit, ab150081 or Goat anti-mouse, ab150117, 1:500, Abcam) for 1 h at RT. Cell nuclei were counterstained with 2-(4-Amidinophenyl)-6-indolecarbamidine dihydrochloride (DAPI; Beyotime) for 5 min at 37°C. The images were obtained by confocal fluorescent microscope (LSM710, Carl Zeiss).

### Statistical analysis

Data were expressed as the mean ± standard deviation. Statistical significance was assessed by one-way analysis of variance (ANOVA) followed by Tukey’s *post hoc* analysis. *P *<* *0.05 was considered statistically significant.

## Results and discussion

### Preparation and characterization of GelMA

**Figure 1:**
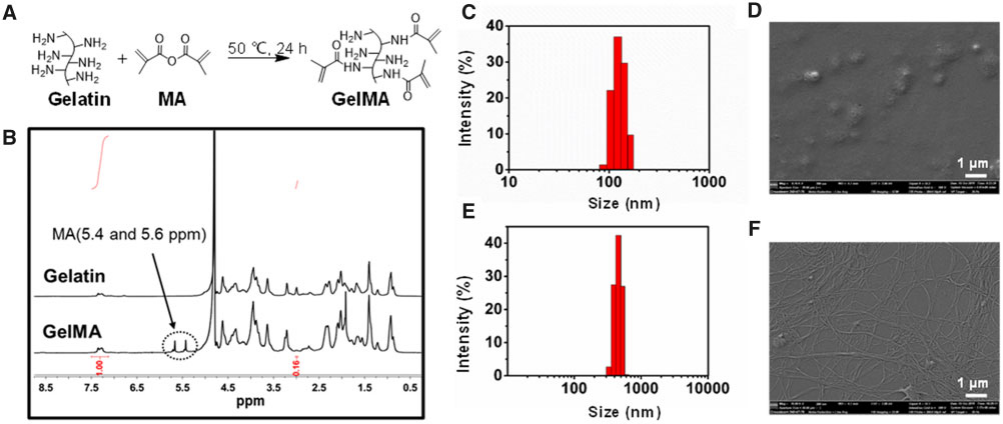
Characterization of methacrylated gelatin (GelMA) and bacterial cellulose (BC) particles. (**A**) Scheme of gelatin methacrylation. (**B**) ^1^H NMR spectra of GelMA and gelatin. (**C**) Size distribution of BC particles. (**D**) Scanning electron microscopy (SEM) of BC particles. (**E**) Size distribution of BC nanofibers. (**F**) SEM of BC particles

In this study, gelatin was modified with MA groups as shown in Fig. 1A. The structure of gelatin and GelMA was determined by ^1^H NMR spectroscopy. Compared with the ^1^H NMR spectrum of gelatin, a new signal appeared at δ 5.4 and 5.6 ppm in the ^1^H NMR spectrum of GelMA, indicating that the methacrylic group was successfully bound to the gelatin (Fig. 1B). The degree of methylation of gelatin could be calculated by the integral ratio between the lysine methylene signal (δ 2.78–2.91 ppm) and the phenylalanine signal (δ 6.99–7.28 ppm) in gelatin [31]. Here, the degree of methacrylation was ∼84%, which was consistent with other reports of GelMA functionalization [32].

### Characterization of BC particles

DLS and SEM were used to evaluate the size and morphology of the obtained BC particles and nanofibers, respectively. As was shown in Fig. 1C, the hydrodynamic size distribution of the particles was around 110 nm. Figure 1D showed that the diameter of BC particles under SEM was larger than the data detected by DLS. The reason is that the edge of the BC particles had a close density to water and could not be sensed by DLS [33]. Figure 1E and F showed that the hydrodynamic size of BC nanofibers was around 500 nm, and the nanofibers were with dispersed distribution.

### Hydrogel formation and morphology

**Figure 2:**
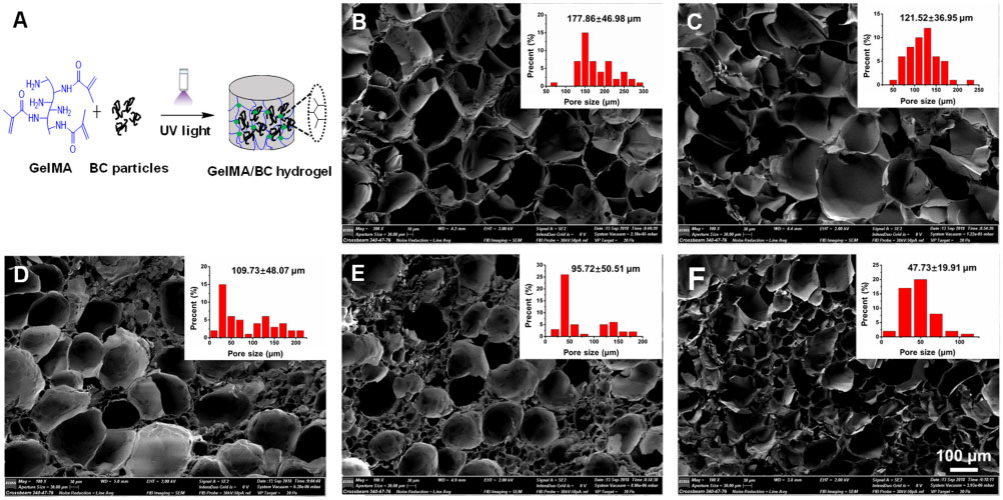
Formation and morphology of the GelMA/BC composite hydrogel. (**A**) Scheme of GelMA/BC hydrogel formation. (**B**–**F**) Cross-sectional SEM images of hydrogels. 0BC (control), 1BC (1 mg/ml), 2BC (2 mg/ml), 4BC (4 mg/ml) and 8BC (8 mg/ml), respectively. Insets were pore size distribution of each hydrogels

GelMA hydrogel was prepared using different UV light initiation times from 30 s to 180 s (Supplementary Fig. S1A). Our results indicated that the rheological properties of hydrogels did not change for exposure times longer than 120 s (Supplementary Fig. S1B), which could be due to the complete cross-linking of the prepolymer solution at 120 s. Therefore, we selected 120 s of exposure time to photopolymerize the hydrogels throughout our study. The GelMA/BC composite hydrogel was fabricated by covalent cross-linking through UV light initiation (Fig. 2A). The cross-sectional SEM images showed that all of the hydrogels had a porous structure with interconnected pores (Fig. 2B–F). The pure GelMA hydrogel (0BC) exhibited a large pore size around 100–200 μm, while the pore size was decreased by introducing BC particles. For 8BC hydrogel, the average pore size was decreased to about 10–100 μm. Those results showed that the average pore size of GelMA/BC composite hydrogels could be adjusted by varying the BC particles content.

With the increase of BC content, more hydrogen bonds were formed inside the GelMA/BC composite hydrogel, which hindered the growth of ice crystals during the freezing process and reduced the pore size and porosity of the hydrogels [34].

### Equilibrium water uptake, XRD and DSC analyses of hydrogel

**Figure 3:**
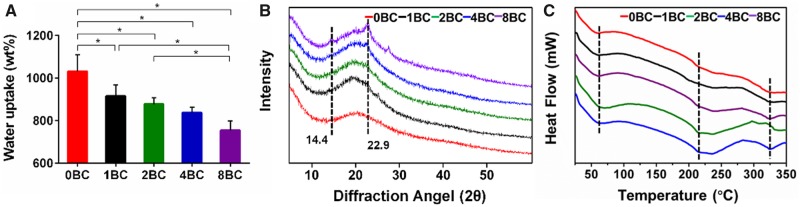
Characterization of the GelMA/BC composite hydrogels. (**A**) Water uptake, (**B**) X-ray diffraction (XRD) curves and (**C**) thermal gravimetric analysis of the hydrogels. Data were presented as mean ± standard deviation (*n* = 3, *P < 0.05)

The water absorption is an important factor to predict the oxygen and nutrient transfer within scaffolds [35]. Figure 3A showed that the freeze-dried GelMA/BC composite hydrogels were swollen in DI water to reach equilibrium water uptake. A decrease in water uptake was observed with introducing BC particles to GelMA hydrogels. The water absorption of pure GelMA hydrogel (0BC) was 1032 ± 78%, however, it decreased to 916 ± 53% when the concentration of BC particles was 1 mg/ml. When the concentration of BC particles increased from 1 mg/ml to 8 mg/ml, the water uptake of GelMA/BC composite hydrogels decreased from 916 ± 53% to 755 ± 44%. The possible reason is that more hydrogen bonds formed in the composite hydrogels with more BC particles, which restricted the hydrogel swelling [33].

A polymer with sharper and stronger XRD peaks shows higher degree of crystallinity, while a relatively weak and wide range of peaks indicates the presence of amorphous regions within the polymer [36]. As shown in Fig. 3B, pure GelMA hydrogel (0BC) presented a less intense peak around 20°, demonstrating the amorphous structure of 0BC hydrogel. Compared with 0BC hydrogel, the GelMA/BC composite hydrogels presented a more intense peak around 20°. What’s more, by increasing the concentration of BC particles (from 1 mg/ml to 8 mg/ml) to 0BC hydrogel, the intensity of peak gradually increased and the maximum sharp intense peak appeared at 22.9°, indicating a higher degree of crystallinity. The crystallinity of BC is due to the presence of hydrogen bonding between the inner layers of polymer [37]. This result indicated that more hydrogen bonds formed in the GelMA/BC composite hydrogels with more BC particles.

DSC was performed to determine the thermal transitions in GelMA/BC composite hydrogels and investigate the effect of BC on the thermal behavior. As shown in Fig. 3C, the DSC curve for all hydrogels gave an endothermic peak at 58°C related to water loss. A broad endothermic peak curve was also observed at 220–300°C, and it could be assigned to the partial pyrolysis due to fragmentation of hydroxyl bonds of the BC [38]. In the case of GelMA/BC composite hydrogels with different concentration of BC particles, the heat flow intensity gradually increased as the increase of the concentration of BC particles, indicating a better thermal stability. The thermal characterization results were in good agreement with the equilibrium water uptake and XRD results.

### Rheological properties of hydrogel

**Figure 4:**
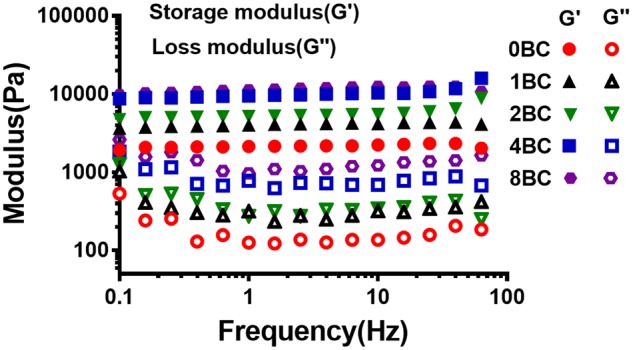
Frequency sweep studies indicating G′/G″ at different hydrogel concentrations

Rheological properties of hydrogel were analyzed via oscillatory rheology to demonstrate the stability of 3D cross-linked networks [39]. Figure 4 displayed the frequency sweep tests of elastic modulus (G′) and loss modulus (G″) within a range of 0.1–10 Hz at fixed strain of 1%. The elastic moduli were larger than the loss moduli, which indicated that the hydrogels displayed a predominantly elastic behavior rather than a fluid-like state. In addition, the rheological properties of hydrogels were dependent on the content of BC particles. The pure GelMA hydrogel (0BC) exhibited a lowest value of G′ about 1.7 × 10^3^ Pa. The G′ values for different GelMA/BC composite hydrogels were about 4.0 × 10^3^ Pa, 5.0 × 10^3^ Pa, 9.0 × 10^3^ Pa and 12.0 × 10^3^ Pa for 1BC, 2BC, 4BC and 8BC, respectively. The rigidity of a hydrogel system network was defined by its G′ values [40]. Those results were attributed to the more stable and dense internal structure of the GelMA/BC composite hydrogels forming a cement mortar structure upon the addition of BC particles.

**Figure 5:**
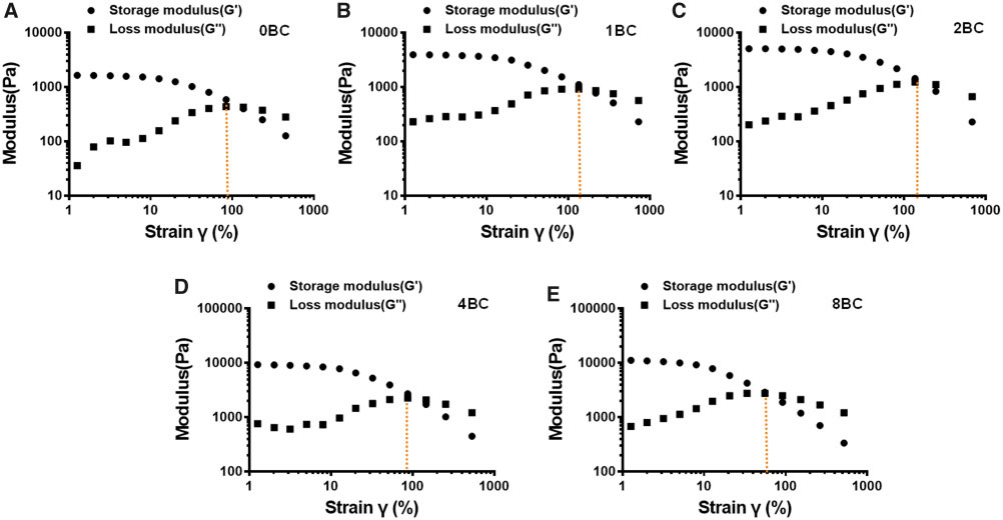
Amplitude sweep studies depicting G′/G″ at different hydrogel concentrations and corresponding crossover values

Amplitude sweep studies were conducted by subjecting the hydrogels to variable strains ranging from 0.1% to 500%. These hydrogels showed signs of crossover around strain values of 100% (Fig. 5). Within 100% strain, a higher BC concentration led to a higher elasticity, which is likely to equip these hydrogels with capability to withstand the administration-related strains as an injectable formulation.

### Mechanical properties of hydrogel

**Figure 6:**
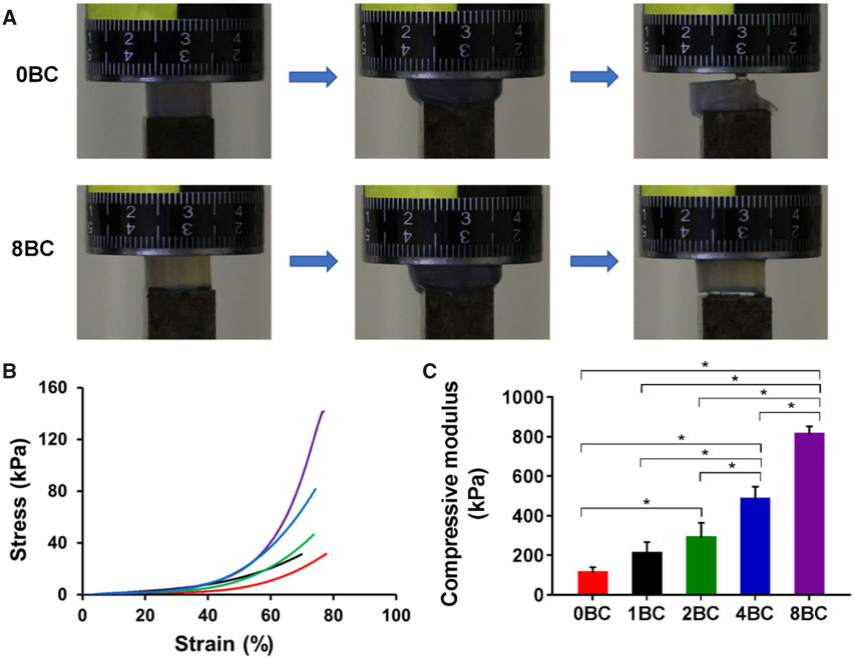
Mechanical properties of the GelMA/BC composite hydrogels. (**A**) Photographs of 0BC and 8BC hydrogels under compression. (**B**) Compressive stress–strain curves. (**C**) Compressive modulus (*n* = 3, *P < 0.05)

Mechanical properties of GelMA/BC composite hydrogels were determined under compression. Figure 6A was the schematic of the compression process of the hydrogels. The 8BC hydrogel could be compressed to 80% strain and its original shape was immediately recovered upon release of the pressure applied on the sample. However, the 0BC hydrogel would be broken under the same compression conditions. This result indicated that the recoverability of hydrogel could be improved by increasing the BC content. From the stress–strain curves (Fig. 6B), compressive modulus, stress-at-failure and strain-at-failure values were determined. The increase of BC content had a great effect on the stress–strain behavior and compressive modulus of GelMA/BC hydrogels. The compressive moduli were determined to be 112.9 ± 15.4, 208.8 ± 33.5, 289.6 ± 43.1, 482.8 ± 37.1 and 811.7 ± 23.4 kPa for 0BC, 1BC, 2BC, 4BC and 8BC hydrogels, respectively (Fig. 6C). Based on these results, the mechanical properties of the GelMA/BC hydrogel could be significantly improved and toughened by increasing the content of BC particles [33]. Overall, GelMA/BC composite hydrogels had tunable compressive moduli ranging from 112.9 ± 15.4 to 811.7 ± 23.4 kPa, in the same range as human articular cartilage [41].

### Cell encapsulation within composite hydrogels

**Figure 7:**
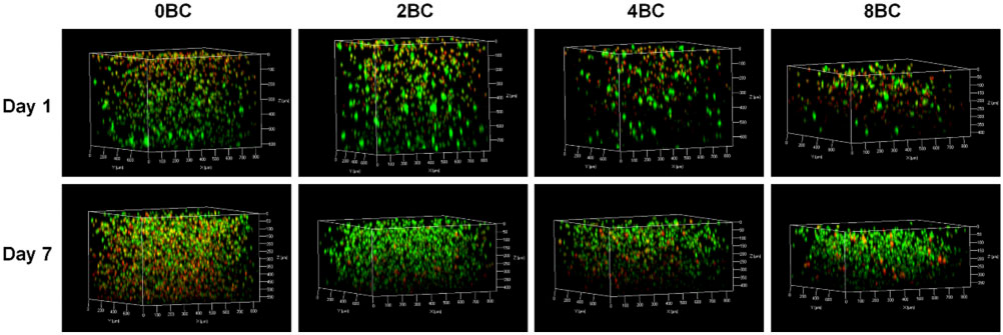
Confocal images showing the live/dead staining of the encapsulated chondrocytes

The material properties of hydrogels significantly influence the behavior of the encapsulated cells. For example, crosslinking density of a hydrogel matrix may affect the cytotoxicity behavior [42, 43]. In this study, the live/dead cell staining results (Fig. 7) showed that, after chondrocyte encapsulation pure GelMA hydrogel (0BC) had a highest initial cell density, while with the increase of BC content, the initial cell density gradually decreased. After 1 week, 0BC group still had the highest cell density, but with a large portion of dead cells; the cell density of other composite hydrogels increased substantially, indicating chondrocyte proliferation and ingrowth within the hydrogels.

**Figure 8:**
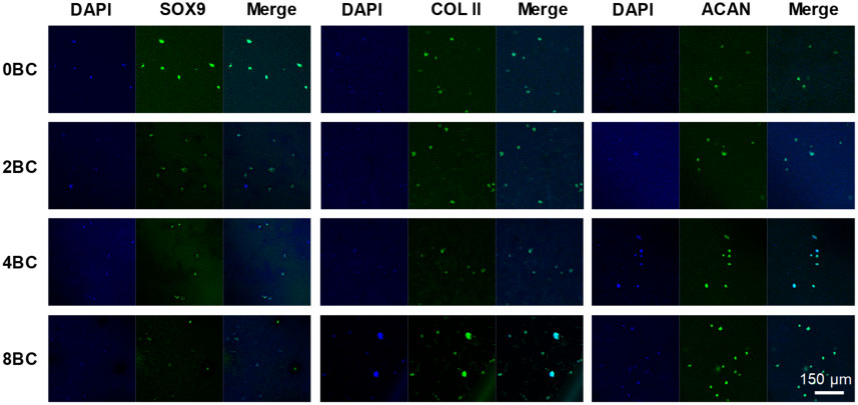
Immunofluorescent staining of encapsulated chondrocytes

In addition to cell proliferation, we also tested the phenotype of these chondrocytes within GelMA/BC composite hydrogels (Fig. 8). After 1 week of encapsulation, the fluorescent staining of chondrogenic markers SOX9, type II collagen and aggrecan were positive in most of the chondrocytes, indicating that the chondrocytes maintained chondrocytic phenotype within the hydrogels.

The limitation of this cell encapsulation study is that the encapsulated chondrocytes were in static-culture. To better mimic the biomechanically dynamic microenvironment *in vivo*, a mechanical load (e.g. cyclic compressiion [44]) or/and chemical chondrogenic agents (e.g. TGF-β1, kartogenin [45]) could be applied to the hydrogel before the measurement of cell proliferation and differentiation.

## Conclusions

BC and GelMA were used to successfully fabricate composite hydrogels using different concentrations of BC. We have determined the material properties of the GelMA/BC composite hydrogels and evaluated the cellular response. With the increase of BC content, the composite hydrogels had smaller pore size and higher mechanical properties. The GelMA/BC hydrogels supported chondrocyte ingrowth, proliferation and phenotype maintenance. The ability to precisely control physical and mechanical properties, and the excellent compatibility with chondrocytes make this composite hydrogel have a good potential in cartilage tissue engineering.

## Funding

This work was supported by the Science Foundation of Guizhou Province ([2019]1428 and [2019]1429).

*Conflict of interest statement*. None declared.

## Supplementary Material

rbz050_Supplementary_Data
