# Supplementary material for: Preparation and characterization of methacrylated gelatin/bacterial cellulose composite hydrogels for cartilage tissue engineering
Source: Regen Biomater. 2019 Dec 19;7(2):195–202. doi: 10.1093/rb/rbz050 (PMC7147361; doi:10.1093/rb/rbz050)
Supplement: rbz050_Supplementary_Data [file rbz050_supplementary_data.pptx]

## Slide 1
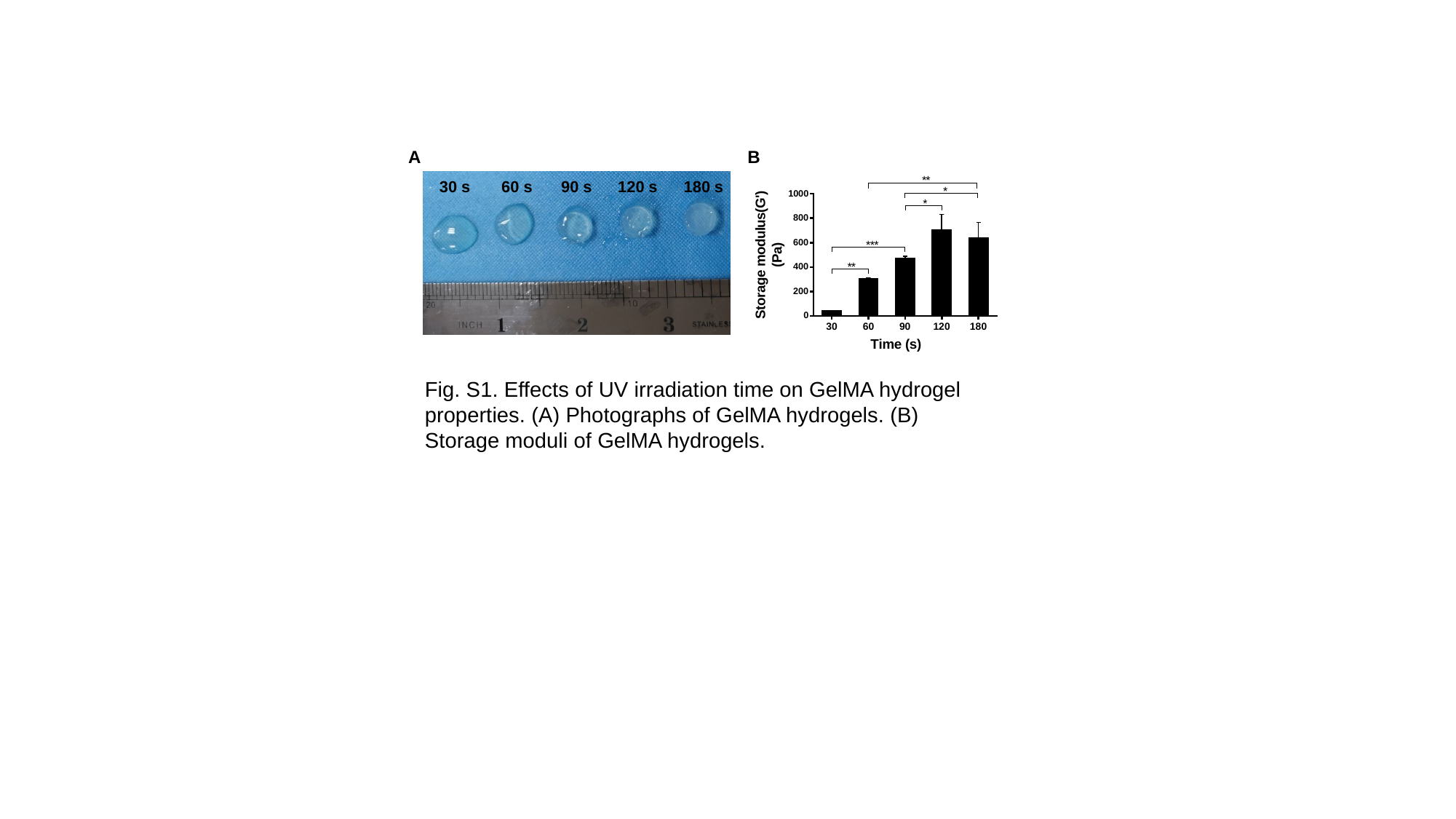

Fig. S1. Effects of UV irradiation time on GelMA hydrogel properties. (A) Photographs of GelMA hydrogels. (B) Storage moduli of GelMA hydrogels.
